# Towards a native environment: structure and function of membrane proteins in lipid bilayers by NMR

**DOI:** 10.1039/d1sc02813h

**Published:** 2021-09-07

**Authors:** Kai Xue, Kumar Tekwani Movellan, Xizhou Cecily Zhang, Eszter E. Najbauer, Marcel C. Forster, Stefan Becker, Loren B. Andreas

**Affiliations:** Max Planck Institute for Biophysical Chemistry, Department of NMR Based Structural Biology Am Fassberg. 11 Goettingen Germany kaxu@nmr.mpibpc.mpg.de land@nmr.mpibpc.mpg.de

## Abstract

Solid-state NMR (ssNMR) is a versatile technique that can be used for the characterization of various materials, ranging from small molecules to biological samples, including membrane proteins. ssNMR can probe both the structure and dynamics of membrane proteins, revealing protein function in a near-native lipid bilayer environment. The main limitation of the method is spectral resolution and sensitivity, however recent developments in ssNMR hardware, including the commercialization of 28 T magnets (1.2 GHz proton frequency) and ultrafast MAS spinning (<100 kHz) promise to accelerate acquisition, while reducing sample requirement, both of which are critical to membrane protein studies. Here, we review recent advances in ssNMR methodology used for structure determination of membrane proteins in native and mimetic environments, as well as the study of protein functions such as protein dynamics, and interactions with ligands, lipids and cholesterol.

## Abstract 2

Solid-state NMR (ssNMR) is a versatile technique that can be used for the characterization of various materials, ranging from small molecules to biological samples, including membrane proteins, as reviewed here.

## Graphical abstract

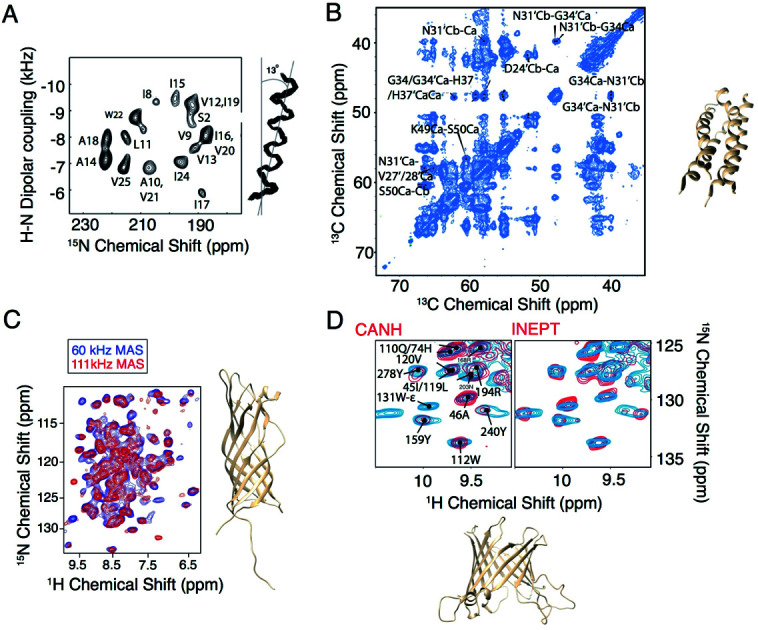

## 1. Introduction

Membrane proteins (MPs) at the interface of cells and the cellular environment, or at the barrier separating cellular compartments, conduct a vast number of functions, such as signal transduction, metabolite transportation and energy conversion.^1^ Due to their varied and important roles, MPs are currently the majority of drug targets.^2^ Understanding the structure and function of MPs is crucial for drug development, but determining their structure and motions remains difficult. Bottlenecks include the difficulty to express them in large amounts in a physiologically relevant state including post-translation modifications, the lack of refolding protocols ensuring the proper folding of the protein in micelles or membranes, as well as the low stability of some MPs.^3^ Major characterization techniques for MPs are X-ray diffraction, cryogenic electron microscopy (cryo-EM) and NMR. No single technique, however, is effective across the wide array of different MPs, and there are often significant discrepancies between structures determined by different methods if the protein's environment was far from a native lipid bilayer.^4^

X-ray diffraction is capable of determining protein structure at a resolution higher than 2 Å, and time resolved crystallography broadens its applicability from static structure determination to investigating dynamic aspects of protein function.^5^ Obtaining high quality crystals in the presence of detergent or lipid cubic phase remains challenging, however.^6^ Advances in single particle cryo-EM has led to an explosion in the number of structures available for large proteins (>50 kDa) and protein complexes. More than 35% of the total membrane protein structures in the Protein Data Bank had been solved by cryo-EM as of 2020. This technique still has difficulties, however, not only in the structure determination of proteins below ∼50–100 kDa (ref. 7) due to the challenge of aligning multiple images for signal averaging, but also in the study of ligand binding.^8^ Cryo-EM samples are mostly prepared in detergents, although a growing number of studies use nanodiscs containing lipids.^9,10^ The use of detergent may weaken tertiary contacts between the protein molecules, leading to a loss of binding specificity and activity for MPs.^4^ Sample preparation for ssNMR is possible in lipid bilayers, or even in the protein's native membrane, but reduced resolution and sensitivity^4,11–13^ compared with microcrystalline preparations leads to long acquisition times. Recent developments in NMR methods such as proton detection using ultrafast magic angle spinning (MAS) probes,^14–19^ and novel isotope labelling schemes^13,16^ as well as increasing magnetic fields (1.2 GHz spectrometers^20^), provide new tools in addressing the bottlenecks encountered in the study of MPs by ssNMR. This article highlights the method development of ssNMR in analyzing MP structure and function. We show applications of ssNMR in the study of ligand binding, probing MP exposure to water or lipids, and determining protein dynamics.

## 2. Historical perspective

### 2.1 MAS and rotational alignment

One of the first membrane protein structures determined by ssNMR was the structure of phospholamban^21,22^ (PLN) using a uniformly aligned preparation. While spectral assignment of oriented samples can be challenging, often relying on the use of several samples with residue specific labelling, changes in the spectrum have a direct structural interpretation, since the dipolar splitting and chemical shifts are directly dependent upon the orientation angle between protein and membrane. This method therefore gives a direct picture of the structure and the structural changes in the transmembrane domain.

While solid samples lack the line narrowing that occurs in solution due to Brownian motion, coherent MAS averaging can be applied to effectively remove the primary deleterious effects of anisotropic interactions. This, in principle enables the application of ssNMR to larger samples. Fast spinning, however, also removes the dipolar and chemical shift interactions that are measured in oriented samples using polarization inversion with spin exchange at the magic angle (PISEMA) spectra.^21,22^ Instead, structure determination approaches in spinning samples typically rely on measurement of many internuclear distances. Exceptions to this are rotationally aligned samples, where uniaxial rotational diffusion in the membrane provides the alignment, rather than alignment of the bulk sample. Such uniaxial rotation allows the benefits of MAS, for example sequential assignment protocols^15,23^ to be combined with the benefits of orientation restraints, leading to exquisite structural data for large proteins, when suitable conditions can be found that allow the protein to uniaxially diffuse.^24^

**Fig. 1:**
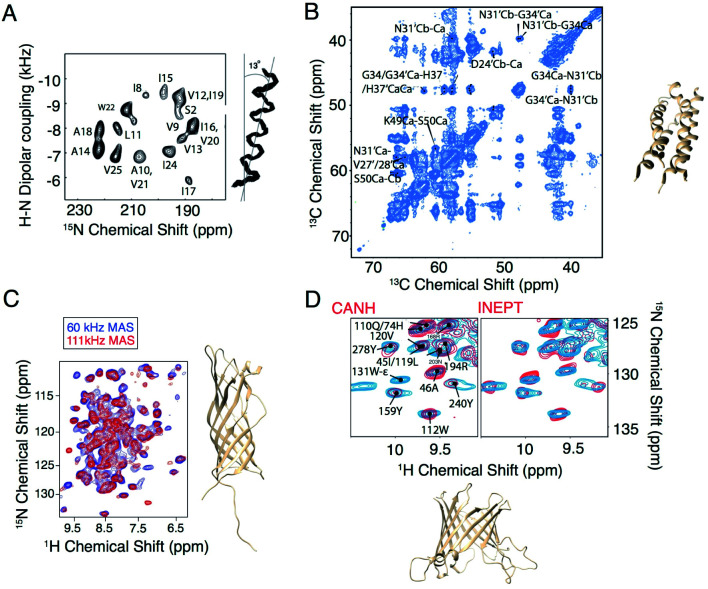
Selected examples of membrane protein structure determination using solid-state NMR. (A) Experimental PISEMA spectrum of uniformly ^15^N labeled trans-membrane ion-channel domain of Vpu in bilayers aligned on glass plates and superposition of 100 calculated backbone structures of the trans-membrane helix of Vpu.^32^ (B) Cα–Cα region of a PDSD spectrum of influenza A M2 S31N with *τ*_mix_ = 400 ms recorded at *ω*_0H_/2π = 750 MHz and *ω*_r_/2π = 14.287 kHz. A 4-fold molar excess of rimantadine drug was present in the sample and did not perturb the chemical shifts of the S31N mutant (code 2N70).^33^ (C) Structure and proton-detected (H)NH spectra of AIkL using 60 and 100 kHz MAS.^27^ (D) Peaks from the Trp side chain region of a CP-based ^1^H–^15^N-correlation (blue) overlaid with either the projection of the (H)CANH spectrum or an INEPT-based sequence (red) and structure of OMPA (code 5MWV).^25^ Panels A and B are reproduced with permission from the American Chemical Society. Panel C is reproduced with permission from Springer. Panel D is reproduced with permission from the Nature Publishing Group.

With the development of faster MAS probes, and the availability of a wide range of pulse sequences for use under MAS to extract structural and dynamic information from membrane protein systems, larger and more complex membrane proteins can be investigated. In the last five years, applications of MAS NMR include the structure determination of OMPG,^25^ HIV gp41,^26^ AIkL,^27,28^ and SARS-CoV-2 envelope protein.^29^ In these applications, membrane proteins are incorporated into lipid bilayers and loaded into ceramic MAS rotors, with the rotor size ranging from 3.2 mm to 0.7 mm in diameter. The packed rotor is spun at the magic angle with the spinning frequency ranging from 10 kHz to more than 100 kHz. At 10–20 kHz, carbon detection is used, while faster MAS reduces the dipolar broadening of protons such that this more sensitive nucleus can be efficiently detected with narrow isotropic chemical shifts. Protein secondary structure is typically determined from the chemical shifts using the program TALOS.^30,31^ Additional information on the protein structure is extracted through dipolar recoupling or polarization mixing sequences that establish the proximity of particular nuclei. This information is combined for structure determination, as shown in Fig. 1. Various dipolar recoupling schemes have been designed, as described in more detail in the next section.

### 2.2 Structures over the years

**Fig. 2:**
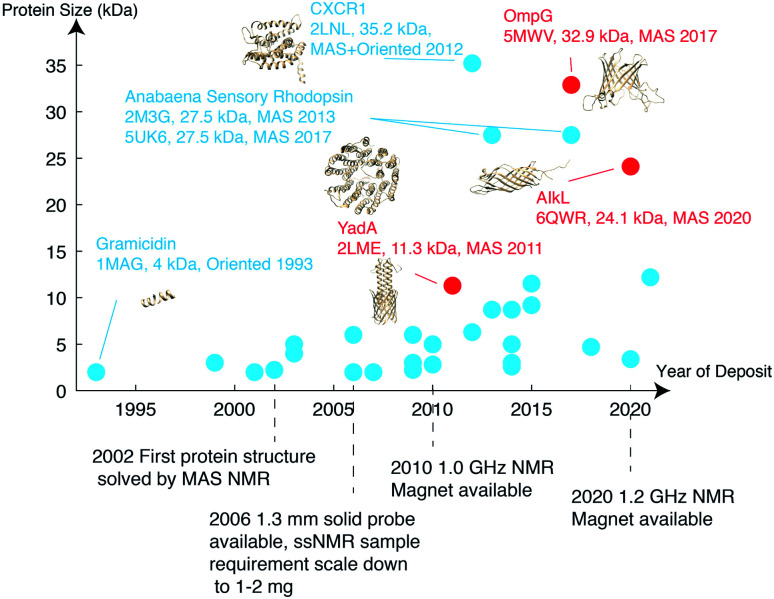
Membrane protein structures deposited in the Protein Data Bank, which were solved by solid-state NMR. Beta barrel structures are denoted in red and helical structures are in blue.

As of 2020, 139 ssNMR protein structures have been deposited in the Protein Data Bank (PDB), of which 56 belong to membrane proteins. Among these, anabaena sensory rhodopsin is the largest with a size of 3 × 27 kDa,^34,35^ characterized using a combination of solid-state NMR and electron paramagnetic resonance (EPR) double electron–electron resonance (DEER) to obtain long-range distances between trimer subunits. When looking only at monomers, the largest membrane protein structure determined belongs to the 35.2 kDa CXCR1.^24^ MP structures deposited since 1993 are listed in Fig. 2 with protein monomer size shown on the vertical axis.

Important new contributions from 2020 include the structures of (I) teixobactins in cellular membranes from Weingarth and coworkers,^36^ (II) outer membrane protein AIkL in DMPC from Andreas, Pintacuda and coworkers,^28^ who also determined the mechanism through which hydrophobic molecules are imported across the beta-barrel AIkL to import hydrophobic molecules across the bacterial outer membrane, (III) the M2 influenza B (M2B) proton channel in the open state, and (IV) the transmembrane domain of the SARS-CoV-2 envelope protein E in ERGIC membranes from Hong's group,^29,37^ including a model for drug-binding. This demonstrates the improving efficiency of NMR techniques in response to urgent biological events like the SARS-CoV-2 pandemic outbreak.

Biological contributions of NMR are enabled by a continued effort in methodology development. In the following sections, we review the progress on this front.

## 3. Methods for structure determination

### 3.1 ^13^C detection-based methods at moderate MAS frequency

Determination of long-range distance restraints by solid-state NMR relies on the synergy of sample preparation schemes and efficient recoupling methods.

#### 3.1.1 Sparse carbon labelling

A limiting factor in using homonuclear dipolar transfers for determining distance restraints in solids is dipolar truncation, which is the phenomenon that a strong dipolar coupling quenches polarization transfer to remote spins. Recoupling schemes, especially first order recoupling schemes transfer more efficiently to the closest spins and this feature limits the possibility of obtaining long-range spin–spin contacts. Recoupling to distant nuclei can be enhanced by sparse labelling. In this approach, target nuclei are isotopically enriched while leaving other nuclei close-by in natural abundance. This can be achieved by using specifically labeled molecules such as glucose or glycerol as the sole carbon source for protein expression. As a result of decreasing the number of NMR active nuclei, spectral overlap is also reduced. This technique has already been applied in both crystalline^38–41^ and membrane protein samples.^34,42–44^ Because of the high required yields for NMR studies, most of these methods have been developed for *E. coli* expression. Meanwhile, eukaryotic expression follows different anabolic pathways. Since some human G-protein coupled receptors either cannot be expressed in *E. coli* or fail to fold in prokaryotic expression systems, protocols to sparsely ^13^C label some eukaryotic membrane proteins have been adapted for expression in *P. pastoris* by Wang and coworkers.^44^

#### 3.1.2 Amino acid specific labelling

Amino acid specific labelling, as indicated by its name, consists of either introduction (forward labelling) or removal of (reverse labelling) all amino acids of certain types. The desired label is usually introduced by addition of amino acids about 1 hour before induction of expression. For forward labelling, other amino acids are typically added at natural abundance to limit conversion of the labeled amino acid to others (scrambling). Scrambling is not a significant concern for the end products of metabolic pathways (*e.g.* Leu, Ile, His), where ^13^C or ^15^N enriched amino acids can be added with no or little scrambling.^45^ Commonly, reverse labelling is done by adding an unlabeled subset of amino acids into a ^13^C or ^15^N enriched labeled medium consisting of ^13^C-glucose and ^15^N-NH_4_Cl, the primary starting molecules of the amino acid metabolic pathway leading to ^13^C, ^15^N enriched amino acids which can later be used by bacteria to synthetize labelled proteins. Addition of an excess of the unlabeled final product can modify the anabolic amino acid pathways, typically limiting amino acid synthesis and allowing efficient incorporation of the unlabeled amino acid. Incorporation of the desired label is heavily dependent on temperature and expression time, which if not controlled, can lead to scrambled labelling. It is also important to note that due to the activity of transaminase, it is possible for the amide ^15^N to scramble but for the ^13^C label to incorporate without scrambling. Amino acid specific labelling schemes are widely used to decrease spectral overlap and provide specific structural information that would be ambiguous using a uniformly labeled sample.^11,25,45^

#### 3.1.3 Recoupling schemes: first and second order recoupling

Distance dependent dipolar couplings are coherently averaged out by MAS, but can be reintroduced using rotor synchronized pulse sequences or through interference with MAS using spin-locking pulses. Rotational echo double resonance (REDOR)^46^ and transferred echo double resonance (TEDOR)^47,48^ are two of the most widely applied heteronuclear recoupling schemes using rotor synchronized pulses. In REDOR, 180° pulses are applied twice per rotor period. In TEDOR, two REDOR periods are applied, sandwiching π/2 pulses on both channels, which induces magnetization transfer in a dipolar analogue to the well-known solution NMR sequences INEPT or HSQC. Early homonuclear recoupling sequences include dipolar recoupling at the magic angle (DRAMA)^49^ and radio frequency driven recoupling (RFDR).^50^ The other class of recoupling sequences, in which dipolar recoupling is achieved with spin-lock pulses, was inspired by the discovery of rotational resonance (*R*^2^)^51^ and rotary resonance recoupling (*R*^3^),^52^ followed by homonuclear rotary resonance (HORROR)^53^ and its adiabatic version dipolar recoupling enhanced by amplitude modulation (DREAM).^54^

Efforts to recouple the Hamiltonian by inspecting the first order term of the Magnus expansion opened up the possibility of measuring heteronuclear and homonuclear distances in isolated spin pairs. The situation in multi-spin systems is more complicated. First order recoupling schemes suffer from dipolar-truncation, and ^13^C–^13^C or ^15^N–^15^N recoupling is influenced by insufficient proton decoupling. Second order techniques are more robust against these limitations. These include proton driven spin diffusion (PDSD),^55^ dipolar assisted rotational resonance (DARR),^56^ mixed rotational and rotary resonance (MIRROR),^57^ phase-alternated recoupling irradiation scheme (PARIS),^58^*R*_2_ driven spin diffusion (RDSD)^59^ and its derivative combined *R*_2_-driven (CORD),^60^ and third spin assisted recoupling (TSAR) sequences including proton assisted recoupling (PAR)^61^/proton assisted insensitive nuclear cross polarization (PAIN-CP),^62^ and a more recent pulsed TSAR.^63^ In spin diffusion, the spin–spin polarization transfer mechanism is explained by the cross-terms between ^13^C–^1^H and ^13^C–^13^C dipolar couplings. In TSAR theory, trilinear terms drive the transfer (X_+/−_X_+/−_H_Z_ or X_+/−_X_−/+_H_Z_ for homonuclear and X_+/−_Y_+/−_H_Z_ or X_+/−_Y_−/+_H_Z_ for heteronuclear transfer).

In a recent study from Hong's group, the structure and drug binding of SARS-Cov2 envelope protein in lipid bilayer was systematically characterized^29^ using CORD and PDSD to obtain ^13^C–^13^C contacts. REDOR was used to extract ^13^C–^19^F distances. ^15^N–^13^C distances were characterized using TEDOR. This work demonstrates the solid-state NMR toolkit for membrane protein studies at moderate MAS (10–40 kHz).

### 3.2 ^1^H detection and fast MAS

When determining long-range distance information, the use of proton–proton distance restraints can be ideal given the fact that proton–proton distances are often shorter than heteronuclear distances in biomolecules, and that protons have a high gyromagnetic ratio. This realization led to the introduction of NHHC and CHHC^64^ which are ^13^C-detected pulse sequences, also used in the study of MP structures. Detection of protons comes with an additional advantage in particular for membrane proteins, since this higher gamma nucleus can be efficiently detected using faster spinning conditions that reduce the amount of sample required.^13,14^ For proton detection at fast MAS frequencies, only about 1 mg or less of sample is required and use of only several labelling schemes or even a single sample can be sufficient due to the information now available from an extra nucleus. Regarding experimental setup in the faster MAS regime (∼40 to 50 kHz and above), RF pulse sequences also differ from the ones applied at moderate MAS. Not only does spin diffusion become less efficient under fast MAS conditions, recoupling schemes are also limited by factors such as the maximal power of the probe, which can be lower than the required multiple of the rotor-frequency. Because of this, the development of additional recoupling schemes with reduced power requirements is still needed for the fast MAS regime. On the other hand, in the fast spinning regime proton decoupling requires only low power, allowing the use longer sequences *e.g.* carbon *J*-transfers.^65^ At fast MAS, double cross polarization (DCP) can be efficiently performed even without proton decoupling.^66^ In 2017, >100 kHz MAS was used to determine a membrane protein structure using protonated proton detection and ^1^H–^1^H RFDR.^16^

#### 3.2.1 Deuteration

Perdeuteration is an approach frequently used to dilute the dense proton network in proteins when MAS is not sufficient to average out the strong homonuclear interactions between the protons. Protons can be reintroduced into perdeuterated proteins through chemical exchange with a protonated solvent. Amide protons in particular provide a sensitive probe at the protein backbone, while perdeuteration of the sidechains eliminates many of the strong proton–proton couplings present in fully protonated samples. Such selective protonation *via* chemical exchange not only improves proton resolution, but the reduced spin diffusion also allows site-specific probing of the proximity of lipids or water. Through back exchange, a clear delineation between hydrophobic transmembrane domains and residues that are only accessible to water can be seen, as detailed further in Section 4.1.^67^ This can offer important structural and functional insights especially for channel membrane proteins with an aqueous pore.

Certain regions of membrane proteins are often protected from chemical exchange, which may be a limiting factor in some cases, but may also be exploited to gain structural information. For example, Ladizhansky's group used this approach to determine the solvent exposed region in proteorhodopsin (PR).^68^ Perdeuterated PR was prepared in a 40% H_2_O/60% D_2_O buffer and 74 out of 254 residues could be detected in the protein for residues where H/D exchange is relatively fast. Since only part of the protein underwent chemical exchange, a global analysis of the perdeuterated protein would require refolding protocols, which are not always available. Differential exchange rates have also been harnessed both to simplify spectra of ion channels, focusing on important residues, and also to address mechanistic questions.^69–72^ A more global view of membrane proteins, while still applying proton detection, can be attained *via* labelling approaches that protonate amides during expression, either with deuterated carbohydrate or amino acid sources,^16,27^ or more simply by using a fully protonated sample in combination with high magnetic fields and fast MAS.^13,16^

**Fig. 3:**
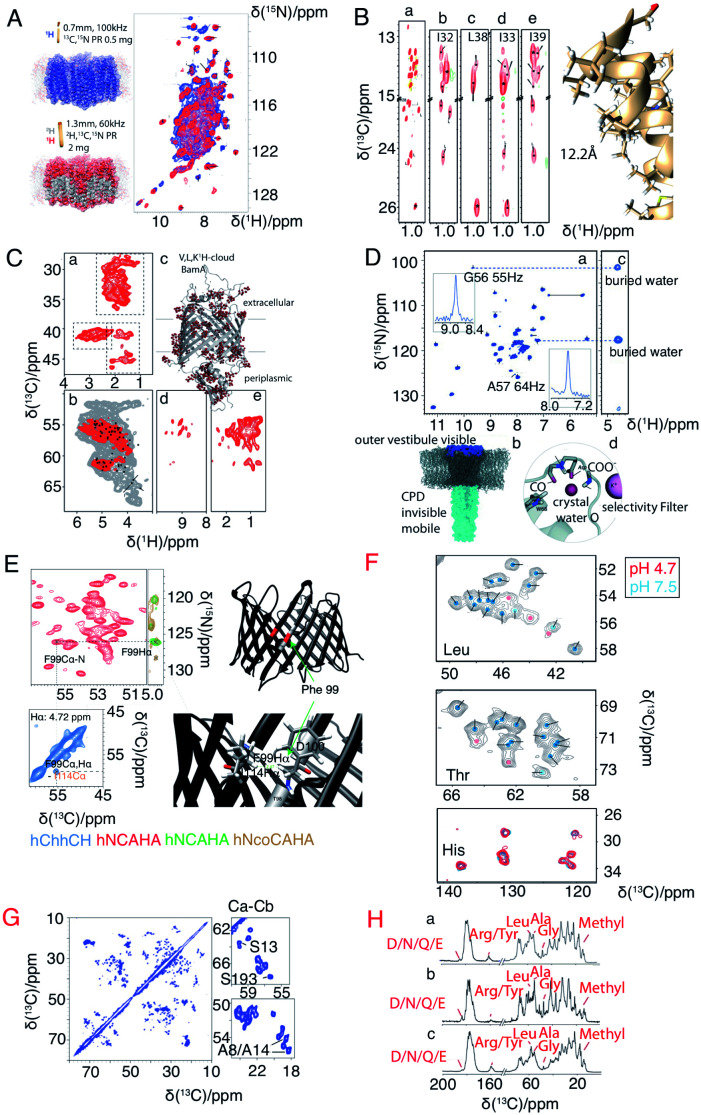
Spectra illustrating protein isotope labelling schemes. Panels A–E show different deuteration schemes for membrane protein samples, and F–H show different carbon labelling schemes. (A) Comparison of 2D ^1^H–^15^N CP-HSQC MAS NMR spectra acquired at 305 K on (blue) fully protonated U-[^13^C,^15^N] proteorhodopsin in DMPC:DMPA lipids at 100 kHz MAS, and (red) U-[^2^H,^15^N,^13^C] proteorhodopsin, reprotonated in 100% protonated buffer, in lipids at 60 kHz MAS and at a field strength of 23.5 T.^16^ (B) Methyl spectra of M2 labeled with –^13^CD_2_H methyl groups in the I, L, and V residues. The *J*-transferred 2D spectrum of the stereospecifically ^13^C^2^H_2_^1^H-methyl-labeled sample is shown in subpanel a, with assignments of isoleucine Cδ1, leucine Cδ2, and valine Cγ2 methyl groups.^33^ (C) Cut-outs of a 2D ^13^C–^1^H spectrum (red) measured at 55 kHz MAS and 800 MHz (sub panel c) using a V,L,K ^1^H-cloud labelling of BamA, exchanged in D_2_O. A spectrum measured with fully protonated BamA (grey) is superposed.^73^ (D) ^1^H-detected (H)NH spectrum of fractionally deuterated KcsA.^74^ (E) Identification of a cross beta strand contact (F99–I114 Hα) in the beta barrel membrane protein VDAC in lipid bilayers.^75^ (F) Resonance assignment of OmpG. Spectral regions of ^13^C–^13^C correlation spectra comprising Cα–Cβ cross-peaks of leucine, threonine, and histidine in three different samples using amino acid specific labelling and DARR mixing. For the peaks indicated by pink dots in these ^13^C–^13^C spectra, no strip could be found in the ^1^H-detected 3D spectra.^25^ (G) ^13^C–^13^C RFDR MAS correlation spectrum of [U-^13^C,^12^C-FLY,^15^N] VDAC in DMPC 2D crystals.^76^ (H) Comparison of sparse ^13^C labelling patterns of rhodopsin from *Leptosphaeria maculans* (LR) in 1D ^13^C-CP MAS spectra. Top to bottom: U-^13^C,^15^N-LR, 2-^13^C-glucose-LR, and 1,3-^13^C-glucose-LR. Significant differences in labelling patterns are marked for methyl groups at 10–20 ppm, Cα of glycine at ∼47 ppm, Cα of Ala at ∼55 ppm, Cα of Leu at ∼58 ppm, Cζ of Arg or Tyr at ∼160 ppm, and carbonyl side chain atoms of Asp, Asn, Glu or Gln at ∼180 ppm.^44^ Panels A–C and G are reproduced with permission from the American Chemical Society. Panel D is reproduced with permission from Wiley. Panel E is reproduced with permission from Springer. Panel F is reproduced with permission from Nature publication group. Panel H is reproduced with permission from Springer.

A major drawback of using perdeuterated proteins is the loss of proton sidechain information. Many research groups have made great efforts to propose optimal methods for the re-introduction of side-chain protons in a controlled manner. One strategy is reduced adjoining protonated (RAP), which uses H_2_O and D_2_O based media for protein expression, which results in a random introduction of protons at each site.^77,78^ Fractional deuteration (FD) uses protonated glucose and a deuterated buffer.^74,79^ Methyl groups, as they are present in structurally important hydrophobic core regions, are especially important in structure determination. Kay's group selectively labelled methyl groups from isoleucine, leucine and valine.^80^ The optimal methyl protonation level at different MAS frequencies was investigated by Reif's group.^81^ With the use of selective –^13^CHD_2_ methyl labelling, Griffin and coworkers extracted ^1^H–^1^H distances in the conductance domain of influenza A M2.^33^Fig. 3 shows a selection of isotopic labeling schemes applied to membrane proteins.

#### 3.2.2 Band selective spectral spin-diffusion (BASS),^82^ mixed rotational and rotary-resonance (MIRROR)^57^

Similar to recoupling schemes for carbon and nitrogen, for proton, methods based on recoupling the second-order Hamiltonian can be more efficient for recoupling distant protons. Spin diffusion is effective only if chemical shift differences are smaller than the zero-quantum dipolar line-width. In the BASS sequence,^82^ a low power spin-lock is applied on the protons, which enhances spin diffusion for spins whose chemical shift difference from the offset position is within the low-power spin-lock range while reducing transfer to spectrally distant spins in the spectra. This method is thus band selective and can specifically detect H_n_–H_n_, H_a_–H_a_, or H_methyl_–H_methyl_ contacts. MIRROR relies on a similar idea, and the irradiation power can be chosen to realize rotational resonance (*R*_2_) and rotary-rotational resonance (*R*_3_) conditions at the same time.^57,83^ In practice, phase modulated and amplitude modulated schemes can be applied instead of continuous-wave irradiation.

#### 3.2.3 TEDOR^84^ and CP

For heteronuclear distance determination, TEDOR and CP are the main tools for magnetization transfer in the fast MAS regime. Determination of distance in membrane proteins is challenged by reduced coherence lifetimes as compared with microcrystalline samples. A recent approach to address this issue is simultaneously recording REDOR and TEDOR (TREDOR),^85^ enabling relaxation-free data interpretation. The method was demonstrated on the Neisserial outer membrane protein Opa60 at a 1.2 GHz spectrometer.

Fast MAS results in better separation of CP conditions. However, ramped or adiabatic shapes in the contact power level still result in incomplete transfer. Ishii and coworkers thus proposed a new concept, decoherence-optimized tilted-angle CP (DOTA CP), where CP efficiency is enhanced by extending ^1^H coherence lifetime under spin-lock. Proton magnetization is flipped by an angle of *θ* between the static magnetic field and the effective field, followed by a CP transfer applying an adiabatic shape on the nitrogen or carbon channel, and finally a 90°–*θ* pulse on proton. About 1.4-fold enhancement was reported in a comparison to adiabatic CP.^86^

Heteronuclear transfer efficiency between the two low-gamma nuclei ^15^N and ^13^C has always been a limiting factor for protein MAS NMR. To improve transfer efficiency, Hong's group proposed proton-enhanced rotor-echo short pulse irradiation cross polarization (PERSPIRATION CP) making use of a second proton spin to transfer magnetization.^87^ Its mechanism is very similar to PAIN-CP but instead of continuous wave irradiation on both channels, rotor echo 90° pulses were used on both the ^13^C and ^15^N channels. In comparison to PAIN-CP, 2-fold weaker RF power is used, and compared to specific-CP, better transfer efficiency is observed.

## 4. Beyond structural studies: solid-state NMR provides unique information about membrane protein environment and mobility

Structure determination of complex biomolecules is often a collaborative effort between crystallography, cryo-EM and NMR. Apart from determining structure, however, NMR can also provide unique information on contacts to flexible species in the membrane environment, on small molecule binding, and on structural dynamics across almost the entire range of timescales.

### 4.1 Contacts to flexible/mobile species such as lipids and water

Solid-state NMR can study membrane proteins in planar lipid bilayers, which is often important for maintaining the structure and function of the target protein. In a protein–lipid system, contacts between the protein and mobile lipid species as well as water are difficult to capture with crystallography and cryo-EM. Solid-state NMR, however, can provide unique information about solvent accessibility and membrane association.^88–90^ Recent examples at fast MAS using proton detection include the human voltage dependent anion channel (VDAC), the alkane transporter AIkL from *Pseudomonas putida*^67^ and influenza A M2 ^91^. Transfer mechanisms in solid-state include the nuclear Overhauser effect (NOE) and spin diffusion, however, in the fast spinning regime, especially when using deuterated samples, spin diffusion is largely suppressed and magnetization transfer occurs mainly through NOE.^67,92^ Both water and lipid correlations were measured for VDAC and AIkL. In addition to lipids/water, cholesterol was recently located on the outside of the beta barrel.^93^ From fitting the buildup of protein–lipid/water cross peak intensity, characteristic cross-relaxation times in the range of seconds were measured. These rates also indicate that transfer to lipids must be considered when determining protein mobility from proton *T*_1_ times.

Novel hardware development like fast spinning probes and high magnetic fields have significantly increased nitrogen *T*_2_ times without the need for high-power decoupling. This enables the detection of weak interactions, as demonstrated in the study of hydrogen bonding among histidine residues in influenza A M2.^91^ The N–H–N hydrogen bond was detected through a weak nitrogen–nitrogen *J* coupling requiring long, ∼40 ms, spin-echo times.

### 4.2 Binding studies

**Fig. 4:**
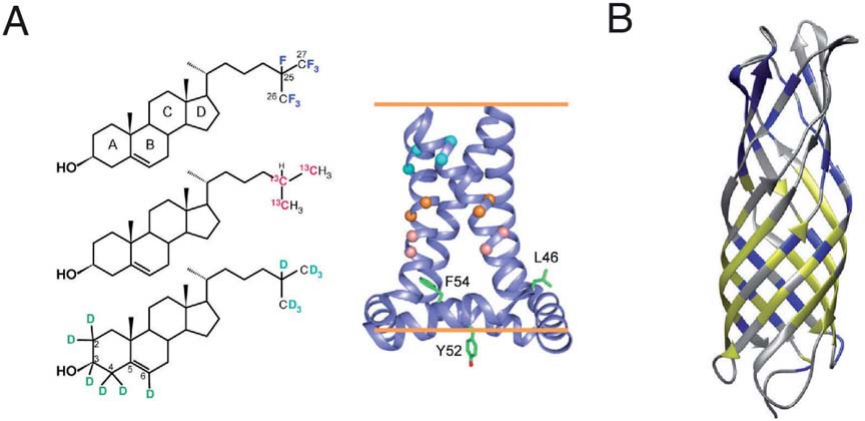
Signal initiated from cholesterol, lipids and water instead of protein. (A) Cholesterol labeled with ^19^F, ^13^C, and ^2^H for determining cholesterol binding to M2. ssNMR structure of Udorn M2(22–62) (PDB ID code 2L0J),^94^ (B) Water and lipid contacts shown on the homology model of AIkL using OmpW (PDB 2F1T) as a template. Lipid contacts are colored yellow and water contacts are colored blue. Residues for which no contact is observed or assigned are colored in grey.^67^ Panel A is reproduced with permission from PNAS, Panel B is reproduced with permission from Wiley.

Two unique advantages make solid-state NMR especially suitable for the study of membrane protein ligand binding: the possibility of investigating ligand binding in a near-native lipid environment, and the strength of ssNMR in clear elucidation of binding kinetics and geometries. Hong and coworkers determined the distance in the M2-cholesterol interaction with ^19^F–^13^C REDOR using cholesterol selectively labeled with ^19^F on the cholesterol aliphatic tail. To determine the orientation of the cholesterol molecule, the sterol head was deuterated and orientation was derived from deuterium quadrupolar couplings. Measurements were performed at 100 K to ensure rigidity of the cholesterol molecule and the signal was enhanced using dynamic nuclear polarization (DNP) hyperpolarization. This work clearly showed that M2-cholesterol interaction does not require the presence of a cholesterol recognition amino acid consensus (CRAC) motif.^94^Fig. 4 shows examples of protein interaction studies with cholesterol, water, and lipids.

The GPCRs containing transmembrane domains interact with many ligands. The neuropeptide Y (NPY) binds to the GPCR Y1R and activates nutrient uptake. NPY's binding sites to the receptor Y1R was determined using chemical shift derived information. ^13^C–^13^C single quantum-double quantum spectra were measured and chemical shift information was analyzed using Rosetta-based molecular docking.^95^

**Fig. 5:**
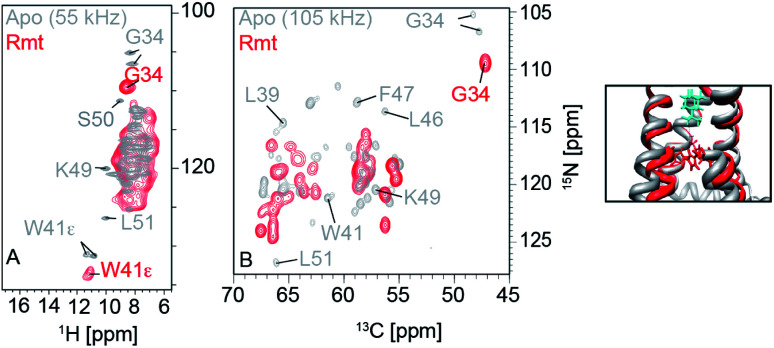
Influenza A M2 HN (left) and NC (right) spectra. Measurements were performed at 950 MHz and 55 kHz or 100 kHz MAS. The apo state is displayed in grey and the rimantadine drug bound state is displayed in red. Large chemical shift perturbations are observed upon drug binding.

Thanks to the sensitivity enhancement and the frozen state, dynamic nuclear polarization (DNP) NMR is especially suitable to study weak and dynamic small molecule interactions with membrane proteins. The inhibition of influenza A M2 by amantadine (AMT) and rimantadine (RMT) was characterized in the weak binding (μM) regime. For these experiments, low temperature was crucial in quenching the mobility of the spherical small molecule in the bound state. When using NOE to detect the binding site, the exchange term commutes with Hamiltonian, but for TEDOR-type of measurements using transverse mixing, the exchange term does not commute,^96^ allowing the use of TEDOR-type measurement at low temperature. Fig. 5 shows chemical shift perturbations that occur due to drug binding in M2.

### 4.3 Dynamics

Protein dynamics are often crucial for understanding protein function.^97,98^ NMR provides information on protein dynamics for almost all timescales of motion, from seconds to nanoseconds. NMR also offers the possibility to measure residue-specific dynamics information simultaneously for thousands of protein observables. Dynamics observables include isotropic chemical shifts, chemical shift anisotropies, dipolar couplings, scalar couplings and quadrupolar interactions for spin >1/2 nuclei. Relaxation times report on dynamics on different time scales.^99–101^

In the past decades, measurement of μs–ms motion (“intermediate to slow” motion on the NMR time scale) has been discussed increasingly in solid-state NMR. The most popular method for characterization of motion on this timescale is relaxation dispersion,^102,103^ which relies on quantifying the line broadening caused by conformational exchange. However, not all conformational change will lead to differences in chemical shifts. Differences in two conformations can also be just a variation of bond vectors. NEar rotary resonance relaxation dispersion (NERRD)^102,104^ is the scheme in solids recently proposed for the measurement of motion at the “intermediate to slow” time regime. It was shown in NERRD that the *R*_1*ρ*_ rate constant in solids is not only related to *B*_0_, spin-lock power and MAS frequency, but also to chemical shift variation and bond-vector fluctuation. Thus, NERRD also allows for visualization of chemical shift identical transient states in solids.

An interesting example for the study of μs–ms dynamics is the rhomboid protease GIpG in liposomes reported by Lange's group.^105^ Rhomboid family intramembrane serine proteases form a large protein family, however, the mechanism of the protease remains elusive. At room temperature and under physiological conditions, solid-state NMR spectroscopy of the protease GIpG in liposomes revealed a kink in the central part of the gating helix (TM5) and a uniform conformation in the upper part of TM5. Relaxation dispersion experiments also suggest that TM5 and L4 (loop between TM5 and TM4) are highly dynamic and exchange between an “open” and “closed” conformation. This information is in contrast with X-ray results in detergent micelle crystals, which suggested capping loop L5 (between TM6 and TM5) movement is the crucial conformational change.

## 5. Outlook

### 5.1 1.2 GHz spectroscopy and beyond

**Fig. 6:**
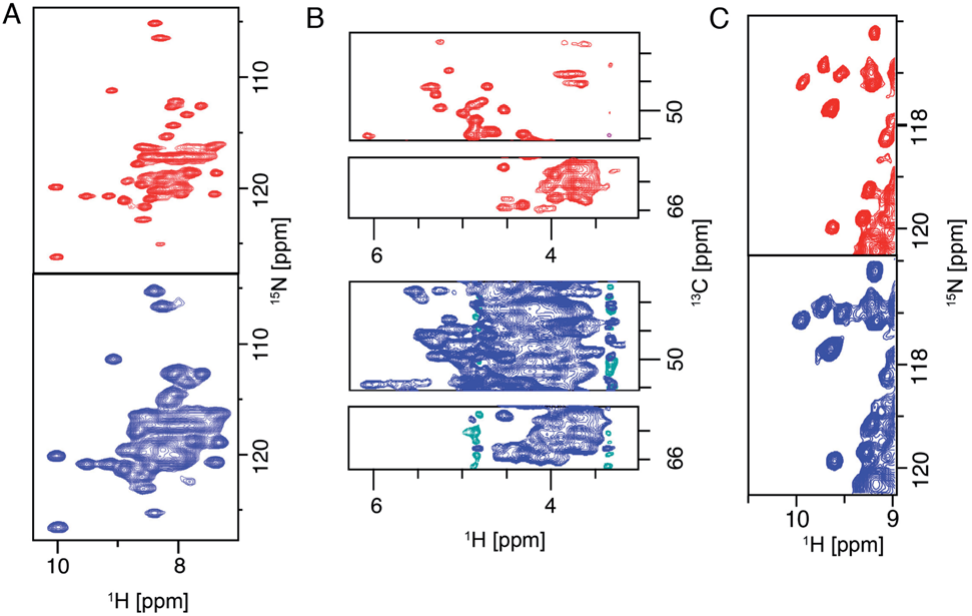
(A) (H)NH spectra of fully protonated Influenza A M2 at 950 MHz and 1.2 GHz spectrometers. (B) Selections from the alpha region of CP-based HC correlation spectra of CitApc. Spectra were recorded on U-[^13^C,^15^N] labelled CitApc reconstituted in protonated lipids. C (H)NH spectra of crystalline α-PET hVDAC. The MAS frequency was 100 kHz and about 0.5 mg of each sample was used. Measurements performed at a spectrometer frequency of 1.2 GHz (28 T *B*_0_ field) are denoted in red and spectra measured at 950 MHz (23.5 T *B*_0_ field) are denoted in blue.^120^ Figures are reproduced with permission from MDPI.

The 28 T magnet (1.2 GHz) went through more than 10 years of development and was awaited with high expectations from the NMR community.^20^ The first commercially available 1.2 GHz magnets were delivered in 2020 to labs in Florence, Zürich and Göttingen.^106^ In the solid state, it has been shown that both resolution and sensitivity of proton-detected experiments are enhanced at high magnetic field.^13,14,17,75,107–109^ A recent study pointed out, even for crystalline proteins using 100 kHz MAS and 1 GHz magnetic field, proton NMR signals are constituted by a broad and a sharp component. Simulation shows that the increase of magnetic field even beyond 1.2 GHz to 2.0 GHz will largely enhance the sharp component which will be beneficial for assignments and analysis.^107^ For more complicated membrane protein samples, this benefit is even more obvious, as shown in Fig. 6 for several membrane proteins. For each of these proteins, the magnetic field improved the linewidth linearly or supralinearly with the increase in field from 950 MHz. Fig. 6B shows spectra of a histidine kinase CitA construct that contains the elements necessary to study signal transduction. For the citrate sensing (PASp), transmembrane, and cytosolic (PASc) domains of CitA, (‘CitApc’, 310 residues per monomer), spectra acquired at 1.2 GHz benefit from the larger chemical shift separation and show more isolated peaks in the HC plane (Fig. 6B). This hints at the possibility for solid-state NMR to be applied to even larger membrane protein systems when even higher magnetic fields become available in the future.

### 5.2 MAS spinning, the faster the better?

The Pintacuda and Böckmann groups, as well as our own group, have shown that proton-detected ultra-fast MAS (>100 kHz) is a powerful tool in the study of membrane proteins.^16,28,110–113^ Meier, Reif, and other groups are showing accumulating evidence that the quality of proton detected spectra continues to benefit from the increase in available spinning frequency.^18,19,81,114–117^ For example, the suppression of homogeneous proton–proton dipolar coupling will still benefit from increasing the MAS rate even beyond 300 kHz,^118^ meaning further hardware development is still important and urgent.^119^

## 6. Conclusions

Solid-state NMR has become a powerful method suitable for determining membrane protein structure, dynamics and function in near-native environments. The number of membrane protein structures determined by solid-state NMR, however, lags behind other methods. In this perspective, we highlighted improvements to the method in terms of hardware and solid-state NMR methodology, prominently, the evolution from detection of carbon, which requires large sample amounts, to proton detection. We addressed key challenges for solid-state NMR applications to membrane proteins, and investigated the possibilities where the strengths of solid-state NMR can be leveraged to study membrane proteins in near-native membrane environments. We highlighted cases where solid-state NMR, by measuring in a near-native membrane environment has resolved controversies arising from conflicting solution and crystal structures. With the availability of 1.2 GHz NMR spectroscopy, probes capable of >100 kHz MAS frequencies, and continued development of pulse sequences for proton detection, we anticipate that in the near future solid-state NMR will become an even more versatile tool for the characterization of membrane proteins.

## Author contributions

K. X., L. B. A. wrote the initial draft, K. T. M., X. C. Z., M. C. F. revised the article, E. N. critically reviewed the article, K. X., M. C. F., K. T. M. and X. C. Z. made figures, S. B. provided membrane protein samples in Fig. 5 and 6.

## Conflicts of interest

There are no conflicts to declare.

## Supplementary Material
